# The ABP Dendrimer, a Drug-Candidate against Inflammatory Diseases That Triggers the Activation of Interleukin-10 Producing Immune Cells

**DOI:** 10.3390/molecules23061272

**Published:** 2018-05-25

**Authors:** Séverine Fruchon, Rémy Poupot

**Affiliations:** INSERM, U1043, CNRS, U5282, Université de Toulouse, UPS, Centre de Physiopathologie de Toulouse-Purpan, F-31300 Toulouse, France; severine.fruchon@inserm.fr

**Keywords:** inflammation, chronic inflammatory diseases, resolution, monocytes/macrophages, interleukin-10, phosphorus-based dendrimers, azabisphosphonate

## Abstract

The ABP dendrimer, which is built on a phosphorus-based scaffold and bears twelve azabisphosphonate groups at its surface, is one of the dendrimers that has been shown to display immuno-modulatory and anti-inflammatory effects towards the human immune system. Its anti-inflammatory properties have been successfully challenged in animal models of inflammatory disorders. In this review, we trace the discovery and the evaluation of the therapeutic effects of the ABP dendrimer in three different animal models of both acute and chronic inflammatory diseases. We emphasize that its therapeutic effects rely on the enhancement of the production of Interleukin-10, the paradigm of anti-inflammatory cytokines, by different subsets of immune cells, such as monocytes/macrophages and CD4+ T lymphocytes.

## 1. Introduction

Inflammation is a physiological process raised by the body in response to any disturbing agent, either of exogenous or endogenous origin. These immuno-stimulatory agents can be living micro-organisms that express at their surface pathogen-associated molecular patterns (PAMPs). It can also be either physically- and chemically-induced damages of a tissue or inappropriately self-modified molecules. These tissue damages and self-modified molecules are referred to as damage-associated molecular patterns (DAMPs). PAMPs and DAMPs are recognized by pattern recognition receptors (PPRs), which are expressed by immunocompetent cells within the circulating blood and the tissues. Most of these cells are professional phagocytic cells, and they are mainly monocytes and their tissue counterpart, macrophages [1]. Following the recognition of PAMPs or DAMPs, the immunocompetent cells elicit downstream signaling cascades leading to the activation of pathways that allow the setup of the inflammatory response. The purpose of inflammation is to eliminate the disturbing element. This is characterized by:

(i) the expression of antigen presenting molecules from the class II major histocompatibility complex (MHC II) and of co-stimulatory molecules (CD80, CD86);
(ii) the secretion of pro-inflammatory cytokines (TNFα, IL1β, IL12…), chemokines (CCL2, CCL5…), nitric oxide (NO), and reactive oxygen species (ROS, such as superoxide anions). All are necessary to eradicate the aggressive agent.

**Figure 1:**
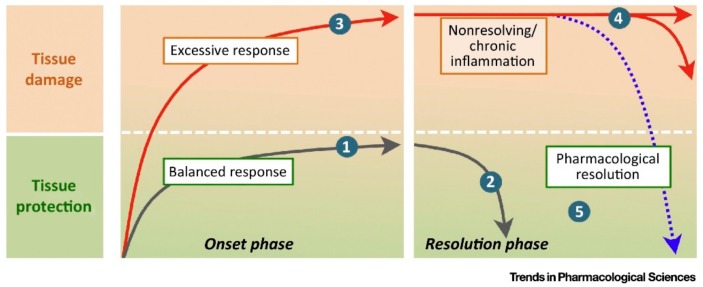
Time phase engagement of proresolving mediators. Inflammation is a physiological response that, when effectively controlled in extent and time, leads to tissue protection without causing tissue damage (profile 1). An effectively mounted inflammatory response will also imply the activation of pathways intended to safely terminate the inflammatory response by ‘cleaning up’ the insulted tissue and promoting healing (profile 2). In some cases, however, an exaggerated (unchecked) response to inflammatory stimuli can have detrimental consequences and result in substantial tissue harm, as in profile 3. A failure in proresolving pathways can extend in time the actions of proinflammatory mechanisms resulting in prolonged (nonresolving) or chronic inflammation (profile 4). It is reasoned that activation of endogenous circuits of resolution through novel resolution-based therapeutics can restore tissue structure and function (return to homeostasis) (profile 5) (taken from [2]) Reproduced with permission from Perretti et al., Trends in Pharmacological Science; published by Elsevier Ltd., 2015.

This effector phase is soon accompanied by the onset and the implementation of the resolution phase (Figure 1, taken from [2]). The aims of this phase are to stop the inflammation once the homeostasis of the infected or damaged tissue has been restored, and to ensure the healing of the tissue [3]. This strict control of inflammation is crucial as persistent unresolved inflammation would lead to chronic inflammatory diseases, including diseased conditions in which inflammation was not thought to be an underpinning mechanism (i.e., Alzheimer’s disease, cardiovascular diseases, and cancers) [3]. The resolution phase of inflammation is characterized by the secretion of so-called pro-resolving mediators, including lipids, peptides and proteins, nucleosides, and gas. These specific mediators trigger cellular and molecular events leading to the control and the resolution of inflammation. Among the cellular events, the reprogramming of monocytes/macrophages from classically-activated (pro-inflammatory, M1 monocytes/macrophages) to alternatively-activated (anti-inflammatory, M2 monocytes/macrophages) cells is a crucial event. This switch then leads to the secretion of anti-inflammatory cytokines, such as interleukin (IL)10 and TGFβ, by the skewed M2 monocytes/macrophages [4].

IL10 is recognized as a major immunomodulatory cytokine which maintains the fine balance between acute inflammation and the regulation thereof. It was discovered by the screening for secreted factors by immunomodulatory CD4+ T helper 2 (Th2) mouse lymphocytes (“anti-inflammatory” cells) that regulate CD4+ T helper 1 (Th1) lymphocytes (“pro-inflammatory” cells) [5,6]. IL10 has a key role in limiting the extent of the inflammatory activation of both the innate and adaptive arms of the immune system. It also regulates the growth and/or the differentiation of several subpopulations of immune cells [7]. Finally, it is produced by a wide variety of immune cells [7], including pro-inflammatory cellular players committed in a strategy of a self-control of the inflammatory response [8]. On the whole, IL10 is a pleiotropic cytokine, with paradoxical effects on different types of immune responses, and particularly involved in physiological feedback loops during inflammation. For instance, the IL10/IL10 receptor (IL10-R) axis is strongly involved in intestinal homeostasis. Consequently, any impairment of the IL10/IL10-R axis, leading to a deficit in IL10 production or signaling, promotes the early onset of colitis in humans [9]. On the contrary, as IL10 is a growth and differentiation factor for B-cells, it is a pathogenic factor in Systemic Lupus Erythematosus (SLE) [9]. Indeed, in this particular auto-immune and chronic inflammatory disease, the level of IL10 in the serum of patients is increased and leads to the enhancement of the production of auto-antibodies by B cells. Logically, the administration of a mouse anti-IL10 monoclonal antibody (aiming at the neutralization of IL10) has shown beneficial outcomes in SLE patients in a small-sized pioneering clinical trial [10]. However, in the most recent reviews in the field, nothing is said about the potential advent of humanized anti-IL10 monoclonal antibodies for the treatment of SLE patients [11,12].

In diseases in which the reinforcement of the IL10 pathway is rationally expected to be beneficial, several therapeutic strategies have been assessed. An obvious strategy is to administer recombinant human (rh) IL10 to patients. Indeed, the subcutaneous injection of rh IL10 has shown a partial therapeutic response in patients with melanoma or renal cell cancer [13]. Rho-associated Kinase (ROCK) 2 is a signaling protein that tunes the concurrent phosphorylation of the Signal Transducer and Activator of Transcription (STAT) 3 (leading to the differentiation of inflammatory Th17 lymphocytes) and STAT5 (leading to the transcription of the *IL10* gene). Inhibition of ROCK2 promotes the phosphorylation of STAT5, therefore the production of IL10. Based thereon, it has been shown that the oral administration of a selective inhibitor of ROCK2 reduces the severity of psoriasis in humans [14]. A third strategy is to transplant autologous M2-like macrophages polarized in vitro from peripheral blood mononuclear cells (PBMC) of the patients. This has been assessed in stroke treatment and has shown a significant improvement of the neurological score of the patients [15].

On the same track, we have discovered and documented the unprecedented immuno-modulatory properties of a particular phosphorus-based dendrimer of the first generation capped with twelve azabisphosphonate (ABP) groups, and that is called the ABP dendrimer. Over the years, we have shown that this unique molecule has anti-inflammatory effects towards several subsets of immune cells (CD4+ T lymphocytes, monocytes/macrophages, and dendritic cells). The anti-inflammatory properties of the ABP dendrimer have been challenged in several animal models of inflammatory diseases, both acute and chronic ones. This mini-review recapitulates the results we have obtained since 2006 and puts in perspective that a common feature of the therapeutic effects of the ABP dendrimer is the enhancement of the production of IL10 via the activation of different subsets of immune cells, namely monocytes/macrophages and IL10-producing CD4+ T lymphocytes.

## 2. The ABP Dendrimer, Birth of a “Lead” Molecule

**Figure 2:**
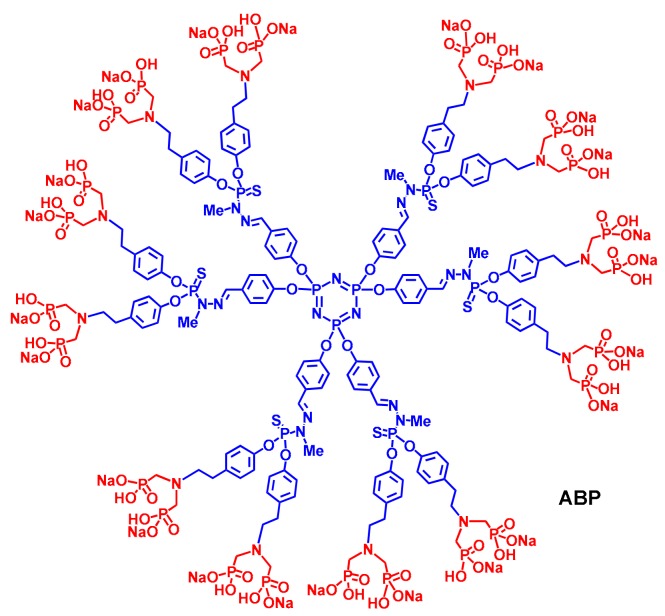
Structure of the ABP dendrimer. The cyclotriphosphazene core (N_3_P_3_) and the PMMH branches (including the point of divergence) are in blue. The twelve tyramine-based (in blue) ABP surface groups are in red.

The ABP dendrimer is built on a cyclotriphosphazene (N_3_P_3_) core on which one series of six phenoxymethyl-methylhydrazone (PMMH) branches are linked. At the end of each branch, the point of divergence is a dichlorothiophosphorus (PSCl_2_) group that enables the doubling of the number branches at the next generation, if any, or the addition of twelve (2 × 6) ABP end groups in the case of the ABP dendrimer (Figure 2).

**Figure 3:**
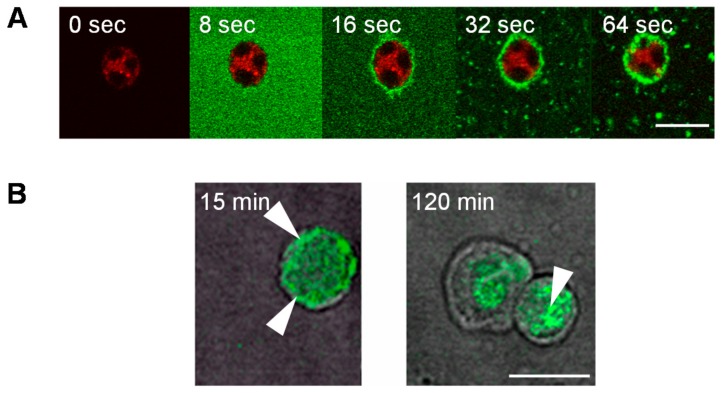
(**A**) Sequential images (64 first seconds) from confocal videomicroscopy of purified monocytes (with cytoplasmic labelling by orange 5-(and-6)-(4-chloromethyl(benzoyl)amino) tetramethylrhodamine [CMTMR]) incubated with the ABP dendrimer emitting green fluorescence, added at second 1. (**B**) Membranous and internal location at 15 min, but only in the intracellular location at 120 min of the ABP dendrimer (white arrows) seen in confocal microscopy; white bars indicate 10 µM (adapted from [19]). Reproduced with permission from Poupot et al., FASEB Journal; published by the Federation of American Societies for Experimental Biology, 2006.

Originally, this molecule was synthesized to activate a particular subset of human T cells, the T Vγ9Vδ2 lymphocytes, which are activated by small pyrophosphate motives [16] and, thereafter, have anti-cancer activity to be used in cancer immunotherapies [17]. As pyrophosphates are easily hydrolyzed in both acidic and alkaline conditions, we have reasoned to replace the phosphonate groups by phosphate ones [18]. Unexpectedly, in preliminary screening studies, the ABP dendrimer has shown its capability to rapidly target (in a few seconds, Figure 3) and efficiently activate human monocytes in vitro [19].

**Figure 4:**
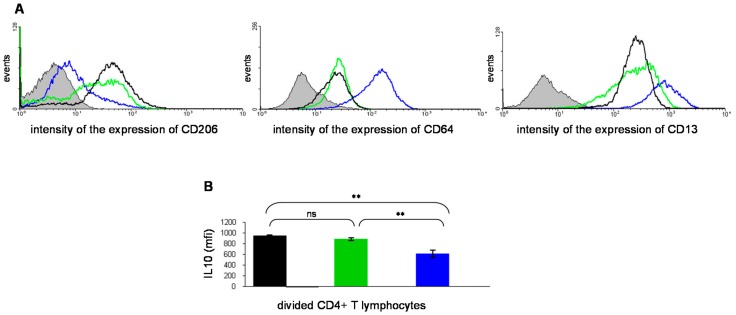
(**A**) Flow cytometry analysis of the phenotype of human monocytes activated in vitro by the ABP dendrimer (black line) compared to M2 (green line) and M1 monocytes (blue line). Left graph: surface expression of CD206 (the mannose receptor MRC1), which is an anti-inflammatory marker; central graph: surface expression of CD64 (a FcγRI receptor), an inflammatory marker; right graph: surface expression of CD13 (the aminopeptidase N), an inflammatory marker. In grey, the negative control of each surface marker (see [22]). (**B**) Flow cytometry quantification (as mean intensity fluorescence, mfi, ± SD) of intracellular IL10 in CD4+ T lymphocytes induced by human monocytes activated by the ABP dendrimer (black bar), by M2 monocytes (green bar) and by M1 monocytes (blue bar) in in vitro co-cultures. ** *p* < 0.001, one-way ANOVA. (see [22]). Reproduced with permission from Fruchon et al., Journal of Leukocyte Biology; published by the Society for Leukocyte Biology, 2009.

As monocytes and macrophages can be activated either towards a pro-inflammatory (the “classical” M1 phenotype [20]) or an anti-inflammatory (the “alternative” M2 phenotype [21]) pathway, we have undertaken the delineation of the mechanism of activation promoted by the ABP dendrimer on human monocytes. For this purpose, in 2009, we completed an unprecedented study of the pan-genomic transcriptome of human monocytes activated in vitro by the ABP dendrimer and compared it to that of resting human monocytes. The analysis of the genes whose expression was up-regulated or down-regulated after exposure of the monocytes to the ABP dendrimer gave a clear-cut answer: the ABP dendrimer promotes an anti-inflammatory response of primary human monocytes in vitro [22]. This result has been confirmed by quantitative real-time PCR on a set of nine genes, among which five code for anti-inflammatory immuno-mediators (MRC1, IL1-RN, IL10, CCL18, and CD23) and four code for pro-inflammatory immuno-mediators (CCL5, IL1β, IL6, and IL12). We have shown that, on one hand, the expression of the five anti-inflammatory genes (especially the expression of the gene coding IL10) was significantly increased in monocytes activated by the ABP dendrimer when compared to resting monocytes. On the other hand, the expression of the pro-inflammatory genes was either decreased (CCL5) or remained unchanged (IL1β, IL6, and IL12). In this study, we have also shown that the phenotype of the monocytes activated by the ABP dendrimer is closely related to the one of anti-inflammatory monocytes (Figure 4A). More interestingly, we have demonstrated that, in mixed leucocyte reactions (MLR), monocytes activated by the ABP dendrimer generate IL10-producing CD4+ T lymphocytes that have immuno-regulatory and anti-inflammatory properties (Figure 4B). These results were the first to show that the ABP dendrimer exerts its anti-inflammatory properties by enhancing the production of IL10 by the human immune cells, both directly by monocytes and indirectly by promoting the appearance of IL10-producing T lymphocytes after activation of the monocytes.

The anti-inflammatory activation of human monocytes has been a test that we have implemented to screen different sets of dendrimers synthesized over the years to depict the relations between the structure and the bio-activity of these molecules. Human monocytes are easily available from blood samples of healthy volunteers. The monocytes are purified from peripheral blood mononuclear cells (PBMC) isolated from the blood by a density gradient. They are then plated in culture wells and cultured for four or five days in the presence of dendrimers in a concentration range of 0.2 to 20 µM. Morphological and phenotypical changes of monocytes are assessed by flow cytometry. Starting from the ABP dendrimer, we have varied four chemical and structural features of dendrimers to uncover the key parameters underpinning the anti-inflammatory activity of dendrimers: the surface functions, the size/generation of the dendrimers, the density of the surface functions at the outer shell of the dendrimers, and the dendritic internal scaffold.

Regarding the surface functions, we have assessed the bio-activity of several monophosphonate [19], azamonophosphonate [19], and symmetric [19] and non-symmetric [19,23] azabisphosphonate groups grafted on the N_3_P_3_ core/PMMH branches of the dendritic scaffold shown in Figure 2 for the ABP dendrimer. This screening reveals that the azabisphosphonate group borne by the ABP dendrimer is the most active. We have also tested isosteric functions of the azabisphosphonate group, namely azabiscarboxylate and azabissulfonate moieties, which turned out to be inactive surface functions [24,25]. Then we evaluated the size, i.e., the generation, and the density of ABP surface functions at the outer shell of the N_3_P_3_/PMMH dendrimers. We have shown that the first-generation dendrimer of this series, with six PMMH branches and twelve ABP groups at its surface (the ABP dendrimer) is the most active, [26]. Interestingly, we have also shown in this latter study that a fluorescent analogue of the ABP dendrimer in which one of the branches is replaced by a fluorescent julolidine group is as bio-active as the ABP dendrimer. This fluorescent analogue is a useful molecular tool to study the mechanisms of action of the “lead” ABP dendrimer [25,27].

**Figure 5:**
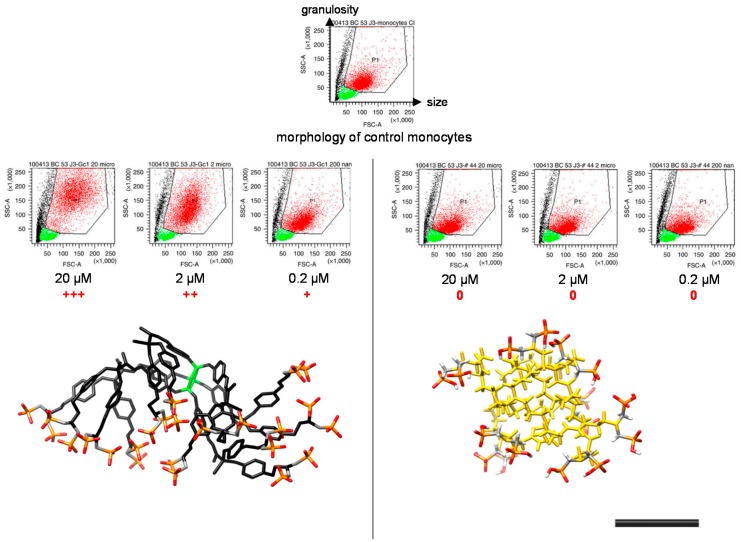
Relation between the bioactivity and the 3D structure of dendrimers. The bioactivity of dendrimers on human monocytes was analyzed by flow cytometry. Each dot in the plots is indicative of morphological changes (size—the Forward SCatter (FSC) parameter on the *x* axis—and granulosity—the Side SCatter (SSC) parameter on the *y* axis) undergone by purified human monocytes (red points gated in the polygon) in the presence of the dendrimers at 20, 2, and 0.2 µM. Green points are the remaining lymphocytes after purification, black points are dead or dying cells. Under the dot plots, the 3D conformation of dendrimers in water solution. +++ indicates strong morphological changes (increase in size and granulosity) when compared to control monocytes, ++ indicates mild changes, + indicates slight changes, 0 indicates no change. On the left: directional conformation of the bio-active ABP dendrimer which is the paradigm of anti-inflammatory ABP-capped dendrimers; on the right: non-directional conformation of an inactive PAMAM dendrimer on the second-generation capped with eight ABP groups at its surface. The black bar represents 1 nm. The phosphonate groups appear in red (oxygen atoms) and orange (phosphorus atoms) (see [28]). Reproduced with permission from Caminade et al., Nature Communications; published by Nature Publishing Group, 2015.

Finally, to understand the role of the dendritic skeleton on the bio-activity of dendrimers, we have prepared a series of thirteen dendrimers with ABP surface groups borne on seven different dendritic scaffolds of the first- and second-generations. The molecular dynamics simulations of these compounds gave an unexpected, but clear, answer: the three-dimensional structure of dendrimers is key to their bio-activity [28]. Indeed, the more that the dendrimers capped with ABP surface groups are directional, the more they are anti-inflammatory (Figure 5). More recently, focusing on the N_3_P_3_/PMMH dendrimers, we have shown that a strong directionality and a low flexibility guarantee a maximization of multivalency, and thereby the anti-inflammatory activity of the molecules [29].

## 3. The ABP Dendrimer, an Immuno-Modulatory New Chemical Entity

Aside from its anti-inflammatory properties towards human monocytes described above, we have shown that the ABP dendrimer has several other immuno-modulatory effects. Very early, we showed that in long-term cultures of human PBMCs, the ABP dendrimer promotes the specific amplification of Natural Killer (NK) cells [30]. NK cells are part of the innate immunity, they patrol in the organism to detect and kill abnormal cells, i.e., infected and cancer cells. That is why for years NK cell-based immunotherapies have been intended to fight against cancers in the clinic [31]. The ABP dendrimer makes it possible to obtain billions of human NK cells ex vivo, the quantity and purity of which are compliant with immunotherapies in humans. Moreover, we have shown that the selective amplification of NK cells in culture with the ABP dendrimer relies on a complex cross-talk between NK cells and dendrimer-activated monocytes [32,33]. On one hand, the latter are mandatory in the culture flask to enable the proliferation of the former, and, on the other hand, the NK cells have to kill dendrimer-activated monocytes before proliferating. The molecular signals involved in this cross-talk (both activating and inhibitory ones) are not yet identified. In the same study [32], we have shown that the N_3_P_3_/PMMH dendrimers capped with ABP groups and identified as bio-active compounds in [26] were able to activate, to some extent, the proliferation of γδ T lymphocytes, a subset of lymphocytes which share phenotypic and functional features with NK cells [34].

**Figure 6:**
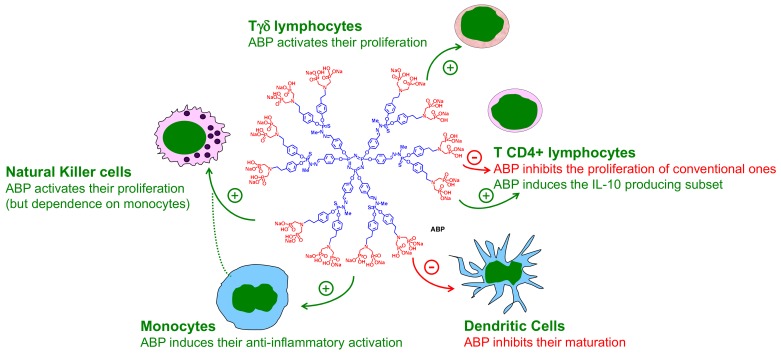
Overview of the different immuno-modulatory effects displayed by the ABP dendrimer towards different sub-populations of human mononuclear immune cells of the peripheral blood: Tγδ lymphocytes [32], T CD4+ lymphocytes [22,35], Dendritic Cells [36], Monocytes [22], Natural Killer cells [30,32].

In addition to the anti-inflammatory activation triggered on monocytes, the ABP dendrimer displays its anti-inflammatory effects towards to other immune populations. Firstly, it is able to inhibit the proliferation of human CD4+ T lymphocytes in vitro [35]. This property can be relevant to treat inflammatory disorders in which the Th1 (i.e., pro-inflammatory) CD4+ T lymphocytes are key players. Secondly, the ABP dendrimer inhibits the maturation of human monocyte-derived dendritic cells in vitro, thereby controlling an inflammatory response [36] (Figure 6). Considering the various anti-inflammatory effects of the ABP dendrimer on human immune cells in vitro, we have challenged it in animal models of inflammatory diseases.

## 4. Proof of Efficacy of the ABP Dendrimer in Animal Models of Inflammatory Diseases

### 4.1. Experimental Arthritis, Mouse Model of Rheumatoid Arthritis (RA)

**Figure 7:**
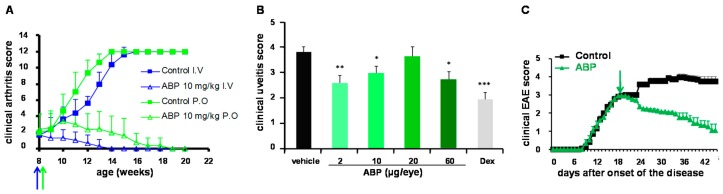
Proof of efficacy of the ABP dendrimer in animal models of inflammatory diseases. (**A**) In a spontaneous mouse model of experimental arthritis, the ABP dendrimer was administered either intravenously (I.V.) or orally (P.O.) at the dose of 10 mg/kg/week starting at the age of eight weeks (blue and green arrows). The clinical arthritis score was monitored weekly ending twelve weeks after the first injection of the ABP dendrimer (maximal score is 12). (**B**) In a rat model of uveitis, the ABP dendrimer was administered loco-regionally (intra-vitreal) once at doses between 2 and 60 µg/eye, the same day than the induction of the disease. The clinical uveitis score was monitored 24 h later (the maximal score is 5). Dex stands for the gold standard dexamethasone at 20 µg/eye in this preclinical model. Histogram bars represent the mean clinical score + SEM. * *p* < 0.05, ** *p* < 0.01, *** *p* < 0.001 versus vehicle. (**C**) In a mouse model of Experimental Auto-immune Encephalomyelitis (EAE), the ABP dendrimer was administered intravenously at the dose of 10 mg/kg/3 days starting 18 days after the onset of the disease (green arrow). The clinical EAE score was monitored daily (maximal score is 5).

The first model we have chosen is a mouse model of experimental arthritis. The mice are genetically modified, namely, knocked-out for the antagonist to the receptor to IL1 (IL1-ra^−/−^) [37]. Therefore, they develop constitutive inflammation and spontaneous arthritis on their four paws starting around the age of four weeks. At eight weeks of age, all the mice are diseased. This preclinical model is considered relevant to human rheumatoid arthritis (RA) as it is dependent both on TNF-α and IL17 [38,39]. The ABP dendrimer was administered weekly to the IL1-ra^−/−^ mice, starting at the age of eight weeks, when the incidence of arthritis is 100%. The molecule was injected intravenously at doses between 0.01 and 10 mg/kg/week, the highest doses at 1 and 10 mg/kg/week being efficient [40]. In this model we have shown that the ABP dendrimer controls the development of the disease and cures its clinical symptoms (Figure 7A). Indeed by targeting and reversing the inflammatory phenotype of monocytes/macrophages to an anti-inflammatory one, the ABP dendrimer enables a dramatic decrease of serum levels of pro-inflammatory cytokines (IL1β, TNF-α, IL6, and IL17) and of matrix metallo-proteinases (MMP-3 and MMP-9) in the serum of treated IL1-ra^−/−^ mice. MMP are responsible for the degradation of joint cartilage, therefore, cartilage is preserved in treated mice when compared to untreated animals. In addition, the ABP dendrimer inhibits both the differentiation of monocytes and the trans-differentiation of myeloid dendritic cells in osteoclasts. These giant multi-nucleated cells are responsible for bone resorption, of which treated IL1-ra^−/−^ mice are thus preserved, contrary to untreated mice. These results have reinforced the relevance of monocytes/macrophages as a cellular therapeutic target for the treatment of RA [41]. Finally, after necropsy of the mice, we have shown that spleen cells from IL1-ra^−/−^ mice treated with the ABP dendrimer for twelve weeks secrete less pro-inflammatory Th1 cytokines (IL1β, TNF-α, IL6, IL17, IL2, and IFN-γ), whereas the secretion of anti-inflammatory Th2 cytokines, such as IL4 and IL10, was dramatically increased, when compared with age-matched untreated IL1-ra^−/−^ mice [40]. Of note, we have discovered that the ABP dendrimer is also active in the IL1-ra^−/−^ mouse model when given per os (orally), solubilized in phosphate buffer without any dedicated formulation, at the dose of 10 mg/kg administered weekly [42]. The therapeutic efficacy of the ABP dendrimer against experimental arthritis has been confirmed in another widely used model of the disease, in which arthritis is induced by the transfer of K/BxN serum (also known as the KRN mouse model) [43]. In this model, inflammation and arthritis are rapidly and transiently induced by transferring serum from autoimmune K/BxN mice into wild-type mice. This model relies on the presence of antibodies specific for the auto-antigen glucose-6-phosphate isomerase, which develop in autoimmune K/BxN mice. We have shown that the ABP dendrimer is active, both in prophylactic and curative protocols, at the dose of 10 mg/kg administered intravenously three times along a setting of eleven days [29,40]. Thanks to these results, the ABP dendrimer has been acknowledged as a promising drug candidate for the treatment of RA [44,45].

### 4.2. Endotoxin-Induced Uveitis (EUI), Rat Model of Anterior Uveitis

Then we have decided to assess the therapeutic efficacy of the ABP dendrimer in a model of acute inflammatory disease cured through topical application of the drug, instead of systemic administration in the above arthritic models. We have chosen Endotoxin-Induced Uveitis (EIU) in the rat, which is considered a relevant model for human anterior uveitis [46]. Uveitis is induced by the systemic administration of LipoPolySaccharide (LPS) which results in an acute inflammatory response in the anterior and posterior segments of the eye with a breakdown of the blood-ocular barrier and infiltration of inflammatory immune cells from the periphery. This model is widely used in preclinical trials for the development of new drugs [47,48]. We have shown that, when administered in the vitreous at 2 µg per eye, the ABP dendrimer is a credible competitor of the gold standard Dexamethasone at 20 µg per eye (Figure 7B) [49]. In particular, we have shown that the ABP dendrimer strongly increases the level of IL10 in the serum of treated EIU rats, as Dexamethasone does. On the contrary, there is no significant decrease of pro-inflammatory cytokines (TNF-α, IL1β, IL2, IL6, IL17, and IFN-γ) nor significant increase of anti-inflammatory cytokines (IL4 and IL10) in the eyes.

### 4.3. Experimental Auto-Immune Encephalomyelitis (EAE), Mouse Model of Multiple Sclerosis (MS)

Experimental Auto-immune Encephalomyelitis (EAE) induced in wild-type mice upon injection of myelin oligodendrocyte (MOG) peptide and pertussis toxin is recognized as a relevant preclinical model of human multiple sclerosis (MS) [50]. As autoreactive pro-inflammatory CD4+ T lymphocytes are key players in the onset and the development of the disease [51,52], it seemed rational and valuable to assess the effect of the ABP dendrimer in an animal model of MS insofar as it is able to inhibit the proliferation of CD4+ T lymphocytes [35]. The efficacy of the ABP dendrimer has been evaluated in both preventive and curative therapeutic protocols in the EAE mouse models. The molecule shows efficient curative effects at 10 mg/kg when administered intravenously every three days (Figure 7C) or weekly [53]. We have shown that it has the same efficacy than the gold standard Fingolimod (FTY720), which is proposed as the first line treatment to patients with relapse-remitting MS. In the same way than in the IL1-ra^−/−^ mouse model of arthritis, we have shown that splenocytes from EAE mice treated with the ABP dendrimer produce significantly lower quantities of IFN-γ and IL17 after ex vivo stimulation with the MOG peptide. Conversely, they produce higher amounts of IL10. To explain these observations, we have unveiled that several cellular mechanisms are operating in vivo in the mouse model of EAE. The ABP dendrimer skews the production of cytokines by splenocytes from diseased mice from an inflammatory pattern to an anti-inflammatory one. It also inhibits the accumulation of antigen-presenting cells (APC) in the spleen under inflammatory conditions, and prevents APC activation. Most interestingly, the ABP dendrimer also redirects the deleterious immune response of MOG-specific CD4+ T lymphocytes towards a benefic production of IL10. This latter effect is, at least in part, indirect through the action of the ABP dendrimer on APC. Taken together, these data strongly suggest that the ABP dendrimer, by down-regulating the activity of APC, could strongly impact the pathogenic profile of autoreactive CD4+ T cells interacting with these APC. Consequently, autoreactive CD4+ T cells produce lesser amounts of Th1/Th17 pro-inflammatory cytokines and are redirected to produce the anti-inflammatory IL10.

## 5. Conclusions

For years dendrimers have been considered promising players of the nanomedicine landscape [54]. However their advent as drug candidates is hampered by technical issues both in the scale-up of synthesis and in the regulatory process of drug development, in particular, implementation of analytical methods and well-characterization [55,56]. So far, only one dendrimer has reached the clinical phase for the topical treatment of sexually transmissible diseases [57]. This microbicide dendrimer based on a polysine scaffold capped by naphtyl di(sodiumsulfonate) has accessed Phase 2 [58]. Several families of dendrimers have shown anti-inflammatory properties [59]. Among them, the ABP dendrimer is the only one that has been evaluated in three different animal models of inflammatory diseases [40,49,53]. As reviewed herein, a common feature of its therapeutic effect is the enhancement of the production of IL10, the paradigm of anti-inflammatory cytokines. Depending on the model, the cells responsible for the production of IL10 can be monocytes/macrophages or CD4+ T lymphocytes. Formerly, we showed that the same cellular subsets among human immune cells were also able to produce IL10 ex vivo upon stimulation by the ABP dendrimer [22,35]. Among the recent studies that have evaluated the anti-inflammatory effects of dendrimers in vivo, only one has reported an increase of the expression of IL10 at the mRNA level [60]. This increase was evidenced both in pancreatic tissue and peritoneal macrophages in mice with acute pancreatitis treated with G5 PAMAM dendrimers. In this article, nothing is said about an increase or not of IL10 at the protein level. On the contrary, in an infectious model of acute gut inflammation, the authors have shown that the therapeutic effects of both PAMAM and optimized polypropyletherimine (PETIM) dendrimers do not rely on an enhancement of the expression of IL10 [61].

Aside from the assessment of the therapeutic efficacy of drug-candidates in relevant preclinical animal models of diseases, the safety of the compounds has to be documented as early as possible. That is the reason why we have performed a general and immuno-safety study in non-human primates [62]. Using a sub-chronic protocol (four intravenous injections of the ABP dendrimer at one week intervals and ten times the lowest active dose in animal models), we have shown that the ABP dendrimer has no irreversible or long-term effects on the dozens of biochemical and hematological parameters that have been tested. We have also demonstrated that the molecule has no immuno-suppressive effect on the immune system of macaques. Finally, at the necropsy of animals, histo-pathological studies of the main abdomen and thoracic organs reveal no deleterious effect of the molecule. These encouraging results pave the way for the ABP dendrimer to enter the regulatory preclinical phase.

## References

[1] Murray P.J. (2017). Immune regulation by monocytes. Semin. Immunol..

[2] Perretti M., Leroy X., Bland E.J., Montero-Melendez T. (2015). Resolution pharmacology: Opportunities for therapeutic innovation in inflammation. Trends Pharmacol. Sci..

[3] Sugimoto M.A., Sousa L.P., Pinho V., Perretti M., Teixeira M.M. (2016). Resolution of inflammation: What controls its onset?. Front. Immunol..

[4] Ortega-Gomez A., Perretti M., Soehnlein O. (2013). Resolution of inflammation: An integrated view. EMBO Mol. Med..

[5] Fiorentino D.F., Bond M.W., Mosmann T.R. (1989). Two types of mouse T helper cell. IV. Th2 clones secrete a factor that inhibits cytokine production by Th1 clones. J. Exp. Med..

[6] Moore K.W., Vieira P., Fiorentino D.F., Trounstine M.L., Khan T.A., Mosmann T.R. (1990). Homology of cytokine synthesis inhibitory factor (IL-10) to the Epstein-Barr virus gene BCRFI. Science.

[7] Ma X., Yan W., Zheng H., Du Q., Zhang L., Ban Y., Li N., Wei F. (2015). Regulation of IL-10 and IL-12 production and function in macrophages and dendritic cells. F1000Research.

[8] Trinchieri G. (2007). Interleukin-10 production by effector T cells: Th1 cells show self-control. J. Exp. Med..

[9] Geginat J., Larghi P., Paroni M., Nizzoli G., Penatti A., Pagani M., Gagliani N., Meroni P., Abrignani S., Flavell R.A. (2016). The light and the dark sides of interleukin-10 in immune-mediated diseases and cancer. Cytokine Growth Factor Rev..

[10] Llorente L., Richaud-Patin Y., Garcia-Padilla C., Claret E., Jakez-Ocampo J., Cardiel M.H., Alcocer-Varela J., Grangeot-Keros L., Alarcon-Segovia D., Wijdenes J. (2000). Clinical and biologic effects of anti-interleukin-10 monoclonal antibody administration in systemic lupus erythematosus. Arthritis Rheum..

[11] Gatto M., Saccon F., Zen M., Bettio S., Iaccarino L., Punzi L., Doria A. (2016). Success and failure of biological treatments in systemic lupus erythematosus: A critical review. J. Autoimmun..

[12] Doria A., Cervera R., Gatto M., Chehab G., Schneider M. (2017). The new targeted therapy in systemic lupus erythematosus: Is the glass half-full or half-empty?. Autoimmun. Rev..

[13] Naing A., Papadopoulos K.P., Autio K.A., Ott P.A., Patel M.R., Wong D.J., Falchook G.S., Pant S., Whiteside M., Rasco D.R. (2016). Safety, antitumor activity, and immune activation of pegylated recombinant human interleukin-10 (AM0010) in patients with advanced solid tumors. J. Clin. Oncol..

[14] Zanin-Zhorov A., Weiss J.M., Trzeciak A., Chen W., Zhang J., Nyuydzefe M.S., Arencibia C., Polimera S., Schueller O., Fuentes-Duculan J. (2017). Selective oral ROCK2 inhibitor reduces clinical scores in patients with psoriasis vulgaris and normalizes skin pathology via concurrent regulation of IL-17 and IL-10. J. Immunol..

[15] Chernykh E.R., Shevela E.Y., Starostina N.M., Morozov S.A., Davydova M.N., Menyaeva E.V., Ostanin A.A. (2016). Safety and therapeutic potential of M2 macrophages in stroke treatment. Cell Transplant..

[16] Espinosa E., Belmant C., Sicard H., Poupot R., Bonneville M., Fournié J.J. (2001). Y2K+1 state-of-the-art on non-peptide phosphoantigens, a novel category of immunostimulatory molecules. Microbes Infect..

[17] Martinet L., Poupot R., Fournié J.J. (2009). Pitfalls on the roadmap to gammadelta T cell-based cancer immunotherapies. Immunol. Lett..

[18] Hayder M., Fruchon S., Fournié J.J., Poupot M., Poupot R. (2011). Anti-inflammatory properties of dendrimers per se. Sci. World J..

[19] Poupot M., Griffe L., Marchand P., Maraval A., Rolland O., Martinet L., L’Faqihi-Olive F.E., Turrin C.O., Caminade A.M., Fournié J.J. (2006). Design of phosphorylated dendritic architectures to promote human monocyte activation. FASEB J..

[20] Mackaness G.B. (1964). The immunological basis of acquired cellular resistance. J. Exp. Med..

[21] Stein M., Keshav S., Harris N., Gordon S. (1992). Interleukin 4 potently enhances murine macrophage mannose receptor activity: A marker of alternative immunologic macrophage activation. J. Exp. Med..

[22] Fruchon S., Poupot M., Martinet L., Turrin C.O., Majoral J.P., Fournié J.J., Caminade A.M., Poupot R. (2009). Anti-inflammatory and immuno-suppressive activation of human monocytes by a bio-active dendrimer. J. Leukoc. Biol..

[23] Marchand P., Griffe L., Poupot M., Turrin C.O., Bacquet G., Fournié J.J., Majoral J.P., Poupot R., Caminade A.M. (2009). Dendrimers ended by non-symmetrical azadiphosphonate groups: Synthesis and immunological properties. Bioorg. Med. Chem. Lett..

[24] Rolland O., Turrin C.O., Bacquet G., Poupot R., Poupot M., Caminade A.M., Majoral J.P. (2009). Efficient synthesis of phosphorus-containing dendrimers capped with isosteric functions of amino-bis(methylene) phosphonic acids. Tetrahedron Lett..

[25] Ledall J., Fruchon S., Garzoni M., Pavan G.M., Caminade A.M., Turrin C.O., Blanzat M., Poupot R. (2015). Interaction studies reveal specific recognition of an anti-inflammatory polyphosphorhydrazone dendrimer by human monocytes. Nanoscale.

[26] Rolland O., Griffe L., Poupot M., Maraval A., Ouali A., Coppel Y., Fournié J.J., Bacquet G., Turrin C.O., Caminade A.M. (2008). Tailored control and optimization of the number of phosphonic acid termini on phosphorus-containing dendrimers for the ex-vivo activation of human monocytes. Chem. Eur. J..

[27] Ielasi F., Ledall J., Anes A.P., Fruchon S., Caminade A.M., Poupot R., Turrin C.O., Blanzat M. (2016). Influence of PPH dendrimers’ surface functions on the activation of human monocytes: A study of their interactions with pure lipid model systems. Phys. Chem. Chem. Phys..

[28] Caminade A.M., Fruchon S., Turrin C.O., Poupot M., Ouali A., Maraval A., Garzoni M., Maly M., Furer V., Kovalenko V. (2015). The key role of the scaffold on the efficiency of dendrimer nanodrugs. Nat. Commun..

[29] Hayder M., Garzoni M., Bochicchio D., Caminade A.M., Couderc F., Ong-Meang V., Davignon J.L., Turrin C.O., Pavan G.M., Poupot R. (2018). Three-dimensional directionality is a pivotal structural feature for the bioactivity of azabisphosphonate-capped poly(phosphorhydrazone) nanodrug dendrimers. Biomacromolecules.

[30] Griffe L., Poupot M., Marchand P., Maraval A., Turrin C.O., Rolland O., Métivier P., Bacquet G., Fournié J.J., Caminade A.M. (2007). Multiplication of human Natural Killer cells by nanosized phosphonate-capped dendrimers. Angew. Chem. Int. Edit..

[31] Rezvani K., Rouce R., Liu E., Schpall E. (2017). Engineering Natural Killer cells for cancer immunotherapy. Mol. Ther..

[32] Poupot M., Turrin C.O., Caminade A.M., Fournié J.J., Attal M., Poupot R., Fruchon S. (2016). Poly(phosphorhydrazone) dendrimers: Yin and yang of monocyte activation for human NK cell amplification applied to immunotherapy against Multiple Myeloma. Nanomedicine.

[33] Poupot M., Fournié J.J., Poupot R. (2008). Trogocytosis and killing of IL4-polarized monocytes by autologous NK cells. J. Leukoc. Biol..

[34] Pont F., Familiades J., Déjean S., Fruchon S., Cendron D., Poupot M., Poupot R., L’Faqihi-Olive F., Prade N., Ycart B. (2012). The gene expression profile of phosphoantigen-specific human γδ T lymphocytes is a blend of αβ T cell and NK cell signatures. Eur. J. Immunol..

[35] Portevin D., Poupot M., Rolland O., Turrin C.O., Fournié J.J., Majoral J.P., Caminade A.M., Poupot R. (2009). Regulatory activity of azabisphosphonate-capped dendrimers on human CD4+ T cell proliferation enhances ex-vivo expansion of NK cells from PBMCs for immunotherapy. J. Transl. Med..

[36] Degboé Y., Fruchon S., Baron M., Nigon D., Turrin C.O., Caminade A.M., Poupot R., Cantagrel A., Davignon J.L. (2014). Modulation of pro-inflammatory activation of monocytes and dendritic cells by aza-bis-phosphonate dendrimer as an experimental therapeutic agent. Arthritis Res. Ther..

[37] Horai R., Saijo S., Tanioka H., Nakae S., Sudo K., Okahara A., Ikuse T., Asano M., Iwakura Y. (2000). Development of chronic inflammatory arthropathy resembling rheumatoid arthritis in interleukin 1 receptor antagonist-deficient mice. J. Exp. Med..

[38] Nakae S., Saijo S., Horai R., Sudo K., Mori S., Iwakura Y. (2003). IL-17 production from activated T cells is required for the spontaneous development of destructive arthritis in mice deficient in IL-1 receptor antagonist. Proc. Natl. Acad. Sci. USA.

[39] Horai R., Nakajima A., Habiro K., Kotani M., Nakae S., Matsuki T., Nambu A., Saijo S., Kotaki H., Sudo K. (2004). TNF-alpha is crucial for the development of auto-immune arthritis in IL-1 receptor antagonist deficient mice. J. Clin. Invest..

[40] Hayder M., Poupot M., Baron M., Nigon D., Turrin C.O., Caminade A.M., Majoral J.P., Eisenberg R.A., Fournié J.J., Cantagrel A. (2011). A phosphorus-based dendrimer targets inflammation and osteoclastogenesis in experimental arthritis. Sci. Transl. Med..

[41] Davignon J.L., Hayder M., Baron M., Boyer J.F., Constantin A., Apparailly F., Poupot R., Cantagrel A. (2013). Targeting monocytes/macrophages in the treatment of rheumatoid arthritis. Rheumatology.

[42] Hayder M., Poupot M., Baron M., Turrin C.O., Caminade A.M., Majoral J.P., Eisenberg R.A., Fournié J.J., Cantagrel A., Poupot R. (2012). Frequency and route of administration in the treatment of experimental arthritis by phosphorus-based dendrimer. Ann. Rheum. Dis..

[43] Kyburz D., Corr M. (2003). The KRN mouse model of inflammatory arthritis. Springer Semin. Immunopathol..

[44] Bosch X. (2011). Dendrimers to treat rheumatoid arthritis. ACS Nano.

[45] Leah E. (2011). Dendrimer drug mends monocytes. Nat. Rev. Rheumatol..

[46] Rosenbaum J.T., McDevitt H.O., Guss R.B., Egbert R. (1980). Endotoxin-induced uveitis in rats as a model for human disease. Nature.

[47] Chen F.T., Liu Y.C., Yang C.M., Yang C.H. (2012). Anti-inflammatory effect of the proteasome inhibitor Bortezomib on endotoxin-induced uveitis in rats. Investig. Ophthalmol. Vis. Sci..

[48] El Zaoui I., Touchard E., Berdugo M., Abadie C., Kowalczuk L., Deloche C., Zhao M., Naud M.C., Combette J.M., Behar-Cohen F. (2015). Subconjunctival injection of XG-102, a c-Jun N-terminal kinase inhibitor peptide, in the treatment of endotoxin-induced uveitis in rats. J. Ocul. Pharmacol. Ther..

[49] Fruchon S., Caminade A.M., Abadie C., Davignon J.L., Combette J.M., Turrin C.O., Poupot R. (2013). An azabisphosphonate-capped poly(phosphorhydrazone) dendrimer for the treatment of endotoxin-induced uveitis. Molecules.

[50] Ben-Nun A., Kaushansky N., Kawakami N., Krishnamoorthy G., Berer K., Liblau R., Hohlfeld R., Wekerle H. (2014). From classic to spontaneous and humanized models of multiple sclerosis: Impact on understanding pathogenesis and drug development. J. Autoimmun..

[51] Noseworthy J.H., Lucchinetti C., Rodriguez M., Weinshenker B.G. (2000). Multiple sclerosis. N. Engl. J. Med..

[52] Hohlfeld R., Dornmair K., Meinl E., Wekerle H. (2016). The search for the target antigens of multiple sclerosis, part 1: Autoreactive CD4+ T lymphocytes as pathogenic effectors and therapeutic targets. Lancet Neurol..

[53] Hayder M., Varilh M., Turrin C.O., Saoudi A., Caminade A.M., Poupot R., Liblau R.S. (2015). Phosphorus-based dendrimer ABP treats neuroinflammation by promoting IL-10-producing CD4+ T. cells. Biomacromolecules.

[54] Wagner V., Dullaart A., Bock A.K., Zweck A. (2006). The emerging nanomedicine landscape. Nat. Biotechnol..

[55] Svenson S. (2015). The dendrimer paradox—High medical expectations but poor clinical translation. Chem. Soc. Rev..

[56] Kim Y., Park E.J., Na D.H. (2018). Recent progress in dendrimer-based nanomedicine development. Arch. Pharm. Res..

[57] McGowan I., Gomez K., Bruder K., Febo I., Chen B.A., Richardson B.A., Husnik M., Livant E., Price C., Jacobson C. (2011). MTN-004 Protocol Team. Phase 1 randomized trial of the vaginal safety and acceptability of SPL7013 (VivaGel^®^) in sexually active young women (MTN-004). AIDS.

[58] https://clinicaltrials.gov/ct2/show/study/NCT00740584?term=SPL7013+and+HIV+Infections&rank=1.

[59] Fruchon S., Poupot R. (2017). Pro-inflammatory versus anti-inflammatory effects of dendrimers: The two faces of immuno-modulatory nanoparticles. Nanomaterials.

[60] Tang Y., Han Y., Liu L., Shen W., Zhang H., Wang Y., Cui X., Wang Y., Liu G., Qi R. (2015). Protective effects and mechanisms of G5 PAMAM dendrimers against acute pancreatitis induced by caerulein in mice. Biomacromolecules.

[61] Teo I., Toms S.M., Marteyn B., Barata T.S., Simpson P., Johnston K.A., Schnupf P., Puhar A., Bell T., Tang C. (2012). Preventing acute gut wall damage in infectious diarrhoeas with glycosylated dendrimers. EMBO Mol. Med..

[62] Fruchon S., Mouriot S., Thiollier T., Grandin C., Caminade A.M., Turrin C.O., Contamin H., Poupot R. (2015). Repeated intravenous injections in non-human primates demonstrate preclinical safety of an anti-inflammatory phosphorus-based dendrimer. Nanotoxicology.

